# Effect of Transcutaneous Electrical Nerve Stimulation on Multidimensional Pain Outcomes After Thoracic Surgery: A Secondary Analysis of a Randomized Controlled Trial

**DOI:** 10.3390/medicina62081543

**Published:** 2026-08-11

**Authors:** Daniel David Álamo-Arce, Daniel López-Fernández, Raquel Medina-Ramirez, Maria del Pilar Etopa-Bitata, María del Pino Quintana-Montesdeoca, Irene García-Rodríguez, Pino Delia Domínguez-Trujillo, Marlene García-Quintana, Jorge L. Freixinet-Gilart

**Affiliations:** 1 Soc-Dig Research Group, University of Las Palmas de Gran Canaria, 35016 Las Palmas, Spain; danieldavid.alamo@ulpgc.es (D.D.Á.-A.); irene.garcia@ulpgc.es (I.G.-R.); 2 Health Science Faculty, University of Las Palmas de Gran Canaria, 35016 Las Palmas, Spain; daniel.lopez@ulpgc.es (D.L.-F.); pinodelia.dominguez@ulpgc.es (P.D.D.-T.); jorgelorenzo.freixinet@ulpgc.es (J.L.F.-G.); 3 Education Sciences Faculty, University of Las Palmas de Gran Canaria, 35016 Las Palmas, Spain; pilar.etopa@ulpgc.es; 4 Mathematic Department, University of Las Palmas de Gran Canaria, 35016 Las Palmas, Spain; mariadelpino.quintana@ulpgc.es

**Keywords:** TENS, thoracic surgery, postoperative pain, affective pain, rehabilitation, physiotherapy

## Abstract

*Background and Objectives*: Postoperative pain after thoracic surgery is a multidimensional experience that includes both sensory, evaluative, and affective components. While transcutaneous electrical nerve stimulation (TENS) has demonstrated benefits for postoperative pain management, its association with affective pain outcomes remains less explored. This study aimed to explore the affective dimension of postoperative pain through a secondary analysis of data from a previously conducted randomized controlled trial. *Materials and Methods*: Data were derived from a randomized controlled trial investigating the effects of TENS on postoperative recovery after thoracic surgery. A total of 109 patients were included and allocated into three groups: experimental (TENS + physiotherapy, n = 37), placebo (sham TENS + physiotherapy, n = 37), and control (physiotherapy alone, n = 35). The main outcome of interest in this exploratory secondary analysis was the affective dimension of pain, assessed using the Pain Rating Index—Affective (PRI-A) subscale of the McGill Pain Questionnaire before and after the 3-week rehabilitation program. *Results*: Improvements were observed across several pain-related variables during the intervention period. For the affective dimension of pain, the experimental group demonstrated greater reduction in PRI-A scores than the placebo and control groups. The mean reduction in PRI-A was 0.97 in the experimental group, compared to 0.27 in the placebo group and 0.57 in the control group (*p* < 0.0001). Between-group comparisons showed moderate to large standardized effect sizes favoring the experimental group, although the absolute magnitude of PRI-A changes was modest. *Conclusions*: This secondary exploratory analysis suggests that TENS combined with physiotherapy may contribute to improvements in the affective dimension of postoperative pain following thoracic surgery. These findings support further investigation of multidimensional pain outcomes within postoperative rehabilitation settings.

## 1. Introduction

Postoperative pain remains one of the most important challenges following thoracic surgery. Despite advances in surgical techniques and perioperative care, patients frequently experience significant pain during the postoperative period, which may impair respiratory function, reduce mobility, delay participation in rehabilitation, and negatively affect overall recovery [1,2,3]. Inadequate pain control may contribute to ineffective coughing, secretion retention, pulmonary complications, and prolonged functional limitations [2,3,4,5].

Transcutaneous electrical nerve stimulation (TENS) is a non-invasive, low-cost intervention commonly used in pain management and rehabilitation. Based on the gate control theory proposed by Melzack and Wall [6], TENS has demonstrated analgesic effects in a variety of clinical conditions and surgical settings [5,6,7,8,9]. Previous studies in thoracic surgery have reported beneficial effects of TENS on postoperative pain intensity, pulmonary function, analgesic consumption, and recovery-related outcomes [1,2,10]. Furthermore, recent evidence supports its role as a safe adjunctive intervention within multimodal postoperative pain management strategies [4,8].

Although TENS has been widely investigated in postoperative care, most studies have focused on traditional outcomes such as pain intensity, pulmonary function, respiratory muscle performance, and analgesic requirement [1,2,4,10]. However, postoperative pain is increasingly recognized as a multidimensional experience involving sensory, evaluative, and affective components [11,12]. Despite this, the affective dimension of pain has received comparatively little attention in patients undergoing thoracic surgery.

The McGill Questionnaire is one of the most widely used multidimensional pain assessment tools, and its Pain Rating Index—Affective (PRI-A) subscale specifically evaluates affective descriptors associated with pain perception [11]. Importantly, PRI-A reflects the affective dimension of pain; it does not constitute a direct assessment of anxiety, depression, or psychological distress using dedicated psychological instruments.

To date, limited evidence has specifically examined the influence of TENS on affective pain outcomes measured with the PRI-A following thoracic surgery. Therefore, this secondary exploratory analysis of a previously conducted randomized controlled trial aimed to evaluate whether adding TENS to a standardized physiotherapy program was associated with changes in the affective dimension of postoperative pain.

Although the parent randomized controlled trial primarily evaluated the effects of TENS on postoperative pain intensity and related clinical outcomes, the multidimensional nature of pain suggests that the affective pain responses may represent a distinct therapeutic target. The affective dimension of the McGill Pain Questionnaire (PRI-A) reflects the emotional appraisal of pain and may provide complementary information beyond pain intensity alone. Despite growing evidence supporting the analgesic effects of TENS, its potential influence on the affective dimension of postoperative pain remains poorly understood, particularly after thoracic surgery. Therefore, the present secondary exploratory analysis was undertaken to specifically investigate whether TENS influences the affective dimension of postoperative pain.

The present study represents a secondary exploratory analysis derived from a previously conducted randomized controlled trial investigating the effects of TENS combined with physiotherapy following thoracic surgery. The aim of this study was to evaluate whether the addition of TENS to a standardized physiotherapy program was associated with changes in the affective dimension of postoperative pain, assessed using the Pain Rating Index—Affective (PRI-A) subscale of the McGill Pain Questionnaire.

We hypothesized that patients receiving TENS combined with physiotherapy would demonstrate greater improvements in PRI-A scores compared with patients receiving sham TENS plus physiotherapy or physiotherapy alone.

## 2. Materials and Methods

### 2.1. Study Design

This study is an exploratory secondary analysis of data obtained from a previously conducted randomized controlled trial investigating the effects of transcutaneous electrical nerve stimulation (TENS) on postoperative recovery in patients undergoing thoracic surgery [13]. This analysis focuses specifically on the affective dimension of pain, assessed using the PRI-A subscale of the McGill Pain Questionnaire. This secondary exploratory analysis was predefined to investigate the affective dimension of postoperative pain, measured using the Pain Rating Index—Affective (PRI-A) subscale of the McGill Pain Questionnaire, because this outcome was not explored in the primary publication of the parent randomized controlled trial. No additional interventions, assessments, or participant recruitment procedures were conducted for the purposes of this secondary analysis. The original randomized controlled trial was conducted and reported in accordance with CONSORT guidelines [14] (File S1).

The PRI-A subscale of the McGill Questionnaire was included among the outcome measures collected in the original randomized controlled trial protocol. The present manuscript represents a secondary exploratory analysis specifically focused on the affective dimension of pain.

In the parent randomized controlled trial, participants were randomly allocated in a 1:1:1 ratio to the experimental, placebo, or control groups using a computer-generated randomized sequence. Allocation concealment was maintained through sequential assignment procedures performed by an independent researcher who was not involved in participant recruitment, outcome assessment, or treatment delivery. The allocation sequence remained concealed until participants had completed the baseline assessment and were designated to their intervention group.

All assessments, procedures, and outcome measures included in the present exploratory secondary analysis were part of the protocol approved for the original randomized controlled trial. No additional interventions, evaluations, or participant recruitment procedures were conducted for the purposes of this secondary analysis.

### 2.2. Participants

This is a prospective, randomized study designed to investigate the effectiveness of TENS as an analgesic technique in patients who have undergone thoracic surgery. The study was approved by the Research Ethics Committee of the Dr Negrín University Hospital of Gran Canaria and was conducted between June 2021 and February 2022, with registration number NCT04964973 in the Clinical Trials Registry on 20 February 2022 [13]. All patients signed an informed consent form. The secondary analysis was conducted in accordance with the original ethical approval and did not require additional consent.

**Figure 1:**
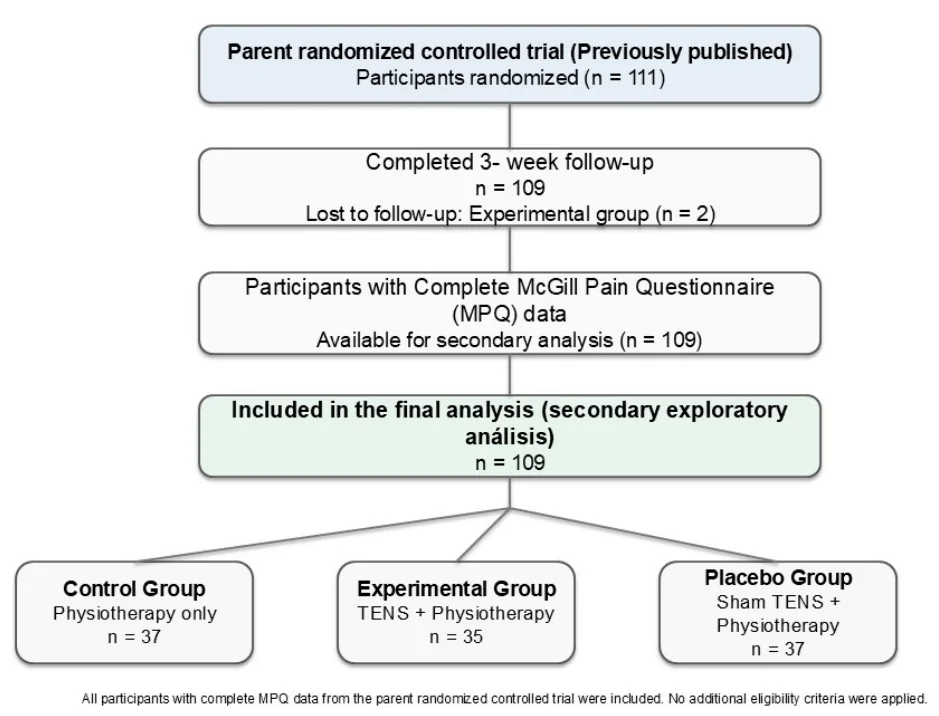
Participant flow for the present secondary exploratory analysis.

Participants were included during the immediate postoperative period once they were clinically stable and able to participate in the physiotherapy program. Surgical procedures were performed within the same thoracic surgery department following standardized institutional perioperative protocols, although not all patients were operated on by the same surgeon. Figure 1 illustrates the flow of participants from the parent randomized controlled trial to the final sample included in the present secondary exploratory analysis. No additional eligibility criteria were applied beyond those specified in the parent trial.

Figure 1 was created by the authors. It is a flow diagram showing participant selection for the present secondary exploratory analysis. The study sample was derived from the parent randomized controlled trial previously published by Álamo-Arce et al. All participants who completed the 3-week follow-up and had completed the McGill Pain Questionnaire (MPQ) data were included in the present secondary exploratory analysis. No additional eligibility criteria or participant selection procedures were applied beyond those defined in the parent trial.

### 2.3. Inclusion Criteria

Patients aged between 18 and 80 years with pulmonary, pleural or mediastinal conditions requiring thoracic surgery via lateral thoracotomy.

### 2.4. Exclusion Criteria

Patients with pacemakers, those with chronic conditions requiring long-term analgesic medication, those with a history of substance abuse, those who required readmission following surgery, those who dropped out of follow-up, and those who did not sign the informed consent form.

### 2.5. Instruments

A McGill Pain Questionnaire (MPQ) was administered to assess the level of immediate post-surgical pain and pain experienced during the therapeutic process. The MPQ measured three main dimensions related to pain: sensory, affective and evaluative. It consisted of 65 words (pain descriptors), grouped into 17 subcategories [15].

The main outcome of interest in this exploratory secondary analysis was the affective dimension of pain, assessed using the Pain Rating Index—Affective (PRI-A) subscale of the McGill Pain Questionnaire. The PRI-A evaluates affective descriptors associated with pain perception and contributes to the multidimensional assessment of the pain experience [15,16].

Following treatment, the MPQ was administered. The differences in these parameters, both before and after treatment, were also measured.

### 2.6. Procedures

#### Physiotherapy Treatment

The standardized physiotherapy program included diaphragmatic breathing exercises, thoracic expansion exercises, respiratory muscle training, early mobilization, and postoperative scar techniques. The same rehabilitation protocol was applied to all groups throughout the intervention period. The intervention also incorporated therapeutic guidance addressing patient’s sensory, emotional, and psychological responses during recovery. This standardized postoperative physiotherapy program was applied to all groups, with TENS or sham TENS added according to group allocation. The physiotherapy program following a standardized institutional postoperative rehabilitation protocol applied consistently across all groups. Baseline assessments were performed during the early postoperative period, once participants were clinically stable and before initiation of the rehabilitation program. Follow-up assessments were conducted after completion of the 3-week intervention period. Emotional and supportive guidance during rehabilitation sessions followed the same general clinical framework across all groups.

In accordance with the proposed randomization, patients were divided into three groups for the application of electrotherapy using TENS, depending on the group [8,17]:

1. Control group. They underwent the standard post-surgical program without the addition of TENS.
2. Experimental group. TENS was added to the physiotherapy program, with a frequency of 100 Hz and a pulse width of 100 µs for a duration of 30 min (via channel 1 of the TENS device), with the patient receiving and feeling the physical sensation of the current.
3. Placebo group. Participants allocated to the placebo group underwent the same treatment procedures as the experimental group, including identical electrode placement, treatment duration, therapist interaction, and visible activation of the TENS device. To simulate the intervention, the device was switched on in the presence of the participant; however, the output channel without electrode cables connected was activated, thereby preventing the delivery of electrical stimulation while preserving the appearance of an active treatment. In addition, participants assigned to the placebo group were scheduled separately from those in the other intervention group and had no contact with participants allocated to the other intervention groups, preventing comparison of treatment experiences and helping participants remain blinded.

TENS was applied using a high-frequency protocol (100 Hz) with a pulse width of 100 µs for 30 min. These parameters are consistent with previous evidence suggesting that high-frequency TENS (50–100 Hz) produces analgesic effects through activation of segmental inhibitory mechanisms and modulation of nociceptive transmission [8,17].

In both experimental and placebo groups, after cleansing the skin with a soapy solution, two adhesive electrodes were positioned parallel to the thoracotomy scar, proximally and distally along the affected intercostal pathway, aiming to modulate nociceptive transmission in the surgical area.

All participants received standard postoperative pharmacological treatment according to the institutional thoracic surgery analgesic protocol. Although minor individual adjustments in medication dosage could be made based on clinical requirements and patient response, postoperative analgesic management remained consistent across groups.

### 2.7. Data Analysis

The sample size calculation was performed for the parent randomized controlled trial using G*Power 3.1. It was based on the overall pain outcome evaluated in the original trial and was designed to detect differences among the three treatment groups, assuming a significance level of 5%, a statistical power of 80%, and an effect size of 0.30. This calculation resulted in a planned sample of 37 participants per group and 111 participants overall. Importantly, the original sample size calculation was not based specifically on the PRI-A outcome. No separate a priori sample size or power calculation was performed for the present secondary analysis. Therefore, the PRI-A analyses should be regarded as exploratory and hypothesis-generating rather than confirmatory.

Given the exploratory nature of this secondary analysis, additional between-group comparisons were performed specifically for the PRI-A subscale to better characterize the magnitude and precision of affective pain differences between interventions. Within the present secondary analysis, PRI-A was considered the primary outcome of interest. Other McGill Pain Questionnaire variables were included to provide descriptive and exploratory contextual information regarding multidimensional pain responses.

IBM SPSS version 27.0 was used for the statistical analysis of the data. Categorical variables were summarized using percentages and absolute frequencies. The non-parametric Binomial and Chi-square tests were used to test whether the proportions across categories were equal. Numerical variables were summarized using the mean and standard deviation. The Kolmogorov–Smirnov and Shapiro–Wilk tests were used to test for normality. Changes in the PRI-A were calculated as the difference between baseline and post-intervention scores. Between-group differences in these change scores were compared using one-way ANOVA or the Kruskal–Wallis test, as appropriate. Given that only two assessment time points were available, this change-score approach was considered suitable for the exploratory nature of this secondary analysis. Effect sizes (Cohen’s d) were calculated for between-group comparisons. Cohen’s d was calculated as the difference between the mean PRI-A change scores of the two groups divided by the pooled standard deviation of the change scores. Effect sizes of approximately 0.2, 0.5, and 0.8 were interpreted as small, moderate, and large, respectively. In addition, 95% confidence intervals were calculated for the between-group differences to quantify the magnitude and precision of the observed effects. Due to the exploratory nature of this secondary analysis, no formal adjustment for multiple comparisons was applied. Therefore, the results should be interpreted with caution, considering the potentially increased risk of Type I error associated with multiple statistical comparisons. In addition to *p*-values, effects sizes and confidence intervals were considered to aid interpretation of the magnitude and precision of observed differences.

The parent randomized controlled trial was reported according to CONSORT guidelines. The present study represents a secondary exploratory analysis of previously collected trial data. The anonymized dataset supporting this analysis is publicly available in Zenodo (https://doi.org/10.5281/zenodo.19707767).

## 3. Results

The present secondary analysis focuses specifically on the affective dimension of pain (PRI-A). The final sample included 109 participants after two patients were lost to follow-up. Baseline characteristics were comparable across groups (Table 1). At baseline, no significant differences were observed between groups. Following the intervention period, improvements were observed across several pain-related variables; however, statistically significant PRI-A reduction was primarily observed in the experimental group. All data used in this analysis are available in the public repository. No significant differences were observed between groups at the baseline. The initial sample comprised a total of 111 patients; two patients were excluded due to loss to follow-up, resulting in a final sample of 109 participants, with a predominance of males (n = 65; 59.6%).

Table 2 summarizes the McGill Pain Questionnaire outcomes before and after treatment for the overall sample. Improvements were observed across several pain-related dimensions during the intervention period. Improvements were observed across several pain-related variables during the study period. These outcomes are presented as contextual and exploratory findings intended to provide a broader characterization of multidimensional pain responses.

Regarding the main outcome of interest, greater reductions in PRI-A were observed in the experimental group compared with the placebo and control groups (Table 3). Between-group comparisons showed a mean difference of 0.40 (95% CI: 0.13–0.67) between the experimental and control groups and 0.70 (95% CI: 0.47–0.93) between the experimental and placebo groups. Corresponding effect sizes were moderate to large (Cohen’s d = 0.68 and 1.17, respectively) (Table 4).

Overall improvements were observed across several pain-related variables during the study period; however, statistically significant improvement in the affective dimension of pain was primarily observed in the experimental group.

## 4. Discussion

This secondary exploratory analysis investigated the affective dimension of postoperative pain in patients undergoing thoracic surgery and receiving transcutaneous electrical nerve stimulation (TENS) as an adjunct to a standardized physiotherapy program. The main finding was that the experimental group demonstrated greater reductions in PRI-A scores than the placebo and control groups following the intervention period. The present secondary exploratory analysis was designed to specifically explore the affective dimension of postoperative pain, an outcome that was collected in the parent randomized controlled trial but not previously analyzed.

Although previous studies evaluating TENS after thoracic surgery have primarily focused on pain intensity, pulmonary function, analgesic consumption, and recovery-related outcomes [13], less attention has been given to the multidimensional aspects of pain. The present findings contribute to this area by exploring the affective dimension of pain using the PRI-A subscale of the McGill Pain Questionnaire.

Pain is increasingly recognized as a multidimensional experience involving sensory, evaluative, and affective components [18,19,20,21]. In the present study, statistically significant differences in PRI-A were observed between groups, with moderate to large, standardized effect sizes favoring the experimental intervention. However, interpretation of these findings should remain cautious because no universally accepted minimally important clinical difference has been established for the PRI-A subscale, and the absolute magnitude of the observed changes was relatively modest. Nonetheless, statistical significance does not necessarily imply clinical significance. Consequently, the present findings should be interpreted as indicative of a potentially relevant change in the affective dimension of pain, rather than as evidence of a clearly established clinically significant improvement. Further studies specifically designed to assess PRI-A outcomes are needed to determine the clinical relevance of changes of this magnitude.

Although the present study analysis focused specifically on the affective dimension of pain, improvements were also observed across additional McGill Pain Questionnaire outcomes. These findings may reflect broader changes in pain experience associated with the intervention and rehabilitation process [4,22,23]. Consequently, the observed PRI-A differences should not be interpreted as evidence of selective modulation of affective mechanisms.

The study population included patients undergoing different thoracic surgical procedures, including lobectomy, segmentectomy, and other resections, which may differ in postoperative pain characteristics and recovery trajectories. However, because this study represents a secondary analysis of a randomized controlled trial, the distribution of surgical procedures across the randomized groups followed the original randomization process, reducing the likelihood of systematic allocation bias. Nevertheless, procedure-related heterogeneity may have influenced postoperative pain responses and should be considered when interpreting the findings. Future studies focusing on more homogeneous surgical populations are warranted to confirm these results.

The inclusion of a placebo group represents an important methodological strength because it helps reduce the influence of nonspecific treatment effects. Nevertheless, participant blinding in sham TENS interventions is inherently challenging because active stimulation produces a perceptible sensory sensation [24,25]. Several procedures were implemented to maximize participant blinding in the present study. The TENS device was visibly activated during placebo treatment, while the output channel without connected electrode cables was selected, preventing electrical stimulation without altering the apparent treatment procedure. Furthermore, participants from different intervention groups were treated separately and therefore could not compare their treatment experiences. The statistical analysis was also performed blinded to treatment allocation. However, because the success of participant blinding was not formally assessed, it cannot be excluded that some participants may have inferred their treatment allocation. This possibility should be considered when interpreting PRI-A, as treatment expectations and contextual effects may influence patient-reported pain outcomes. Similarly, spontaneous postoperative recovery may have contributed to improvements observed across all groups during the study period.

From a clinical perspective, the present findings support the value of multidimensional pain assessment during postoperative rehabilitation. While conventional measures of pain intensity remain important, affective dimensions may provide additional information regarding patients’ pain experience during recovery. However, the clinical relevance of the PRI-A changes observed in the present study remains to be established. Future studies should continue to explore multidimensional pain outcomes and determine whether changes in PRI-A are associated with meaningful improvements in postoperative recovery.

Several limitations should be acknowledged. First, this study represents a secondary exploratory analysis and was not specifically powered to detect differences in affective pain outcomes. The original sample size calculation was not based on PRI-A, and no separate a priori power calculation was performed for this outcome. Therefore, although statistically significant differences were observed, the findings should be interpreted as exploratory and require confirmation in a prospectively designed study specifically powered to evaluate affective pain outcomes. Additionally, the statistical analysis was based on pre–post change scores; although appropriate for the present exploratory analysis with two assessment time points, more flexible longitudinal approaches, such as mixed-effects models, may be considered in future confirmatory studies.

Second, the study was conducted in a single center and included patients undergoing different thoracic surgical procedures, including lobectomy, segmentectomy, and other resections. Although randomization was expected to distribute surgical procedures across groups and thereby minimize systematic allocation bias, this clinical heterogeneity may have influenced postoperative pain responses and may limit the generalizability of the findings to more homogeneous surgical populations. Third, the relatively broad age range of the participants may also limit the generalizability of the findings. Fourth, objective physiological and functional outcomes specifically related to affective pain responses were not available. In addition, although several measures were implemented to maximize participant blinding in the placebo group, formal assessment of blinding success was not performed; therefore, the success of participant blinding cannot be confirmed. Furthermore, physiotherapists were not blinded to group allocation because of the nature of the intervention. Finally, follow-up was limited to the short-term postoperative period, preventing evaluation of longer-term outcomes.

Future research should further investigate multidimensional pain responses following thoracic surgery and explore whether changes in affective pain outcomes are associated with broader rehabilitation and recovery processes. Studies incorporating physiological, functional, and patient-reported outcomes may provide a more comprehensive understanding of postoperative pain experiences and treatment effects.

## 5. Conclusions

This secondary exploratory analysis suggests that transcutaneous electrical nerve stimulation (TENS), when used as an adjunct to physiotherapy, may contribute to improving the affective dimension of postoperative pain following thoracic surgery. Greater reductions in PRI-A scores were observed in the experimental groups compared with placebo and control groups, and these differences were associated with moderate to large standardized effect sizes.

These findings reinforce the importance of considering pain as a multidimensional experience and highlight the potential value of multidimensional pain assessment during postoperative rehabilitation. However, because this study represents a secondary exploratory analysis and the clinical significance of the observed absolute PRI-A changes remains uncertain, the results should be interpreted with caution.

Further research specifically designed to evaluate affective pain outcomes is warranted to confirm these findings and to determine their relevance within broader postoperative rehabilitation and recovery processes.

## Figures and Tables

**Table 1:** Characteristics of the groups studied.

Control Group		Experimental Group	Placebo Group
Diagnosis	Bronchogenic Carcinoma (27)	Bronchogenic Carcinoma (31)	Bronchogenic Carcinoma (22)
	Undiagnosed Pulmonary Nodule (10)	Undiagnosed Pulmonary Nodule (4)	Undiagnosed Pulmonary Nodule (15)
Type of Procedure	Lung Lobectomy (27)	Lung Lobectomy (31)	Lung Lobectomy (22)
	Lung Segmentectomy (10)	Lung Segmentectomy (4)	Lung Segmentectomy (15)
Age	66.27 ± 10.54 (18–80)	64.86 ± 12.14 (18–80)	65.62 ± 10.93 (18–80)
Gender	Men (21)	Men (25)	Men (19)
	Women (16)	Women (10)	Women (18)

Table created by authors.

**Table 2:** Variables relating to the values of PRI-T, PRI-S, PRI-A, and PRI-V.

Variables		Mean (SD)	Mean Difference (SD)	*p*-Value
PRI-S	Start	24.4 (10.2)	6.37 (7.2)	<0.001
	End	18.04 (10.01)		
PRI-A	Start	1.45 (1.2)	0.51 (1.02)	<0.001
	End	0.94 (1.08)		
PRI-V	Start	1.93 (0.89)	0.68 (0.91)	<0.001
	End	1.25 (0.53)		
PRI-TOTAL	Start	27.78 (10.99)	6.64 (7.98)	<0.001
	End	21.14 (10.68)		
PPI	Start	1.88 (0.89)	0.51 (0.94)	<0.001
	End	1.38 (0.57)		
WORDS	Start	17.12 (7.61)	3.65 (5.5)	<0.001
	End	13.47 (7.75)		

Table created by authors. Mean and standard deviation before and after treatment. Abbreviations: total pain rating index (PRI-TOTAL), sensory pain rating index (PRI-S), affective pain rating index (PRI-A), evaluative pain rating index (PRI-V), present pain intensity (PPI); standard deviation (SD).

**Table 3:** Changes in pain-related variables.

Group	Variables		Mean (SD)	Mean Difference (SD)	*p*-Value
Control	PRI-S	Start	27.5 (8.7)	2.6 (4.1)	<0.001
		End	24.8 (7.5)		
	PRI-A	Start	1.3 (0.94)	0.57 (0.76)	<0.001
		End	0.73 (0.8)		
	PRI-V	Start	1.84 (0.87)	0.65 (0.75)	<0.001
		End	1.19 (0.52)		
	PRI-TOTAL	Start	30.59 (8.96)	2.24 (4.37)	<0.001
		End	28.35 (7.61)		
	PPI	Start	2.03 (0.96)	0.405 (0.96)	0.019
		End	1.62 (0.64)		
	WORDS	Start	17.22 (3.6)	0.162 (3.67)	0.096
		End	17.05 (5.2)		
Experimental	PRI-S	Start	21.83 (11)	12.86 (1.33)	<0.001
		End	8.97 (6.7)		
	PRI-A	Start	1.46 (1.4)	0.97 (0.22)	0.001
		End	0.49 (0.92)		
	PRI-V	Start	2.43 (0.82)	1.34 (0.15)	<0.001
		End	1.09 (0.28)		
	PRI-TOTAL	Start	25.71 (12.42)	14.03 (8.71)	<0.001
		End	11.69 (7.53)		
	PPI	Start	1.91 (0.78)	0.77 (0.73)	<0.001
		End	1.14 (0.35)		
	WORDS	Start	14.34 (6.1)	7.2 (5.62)	<0.001
		End	7.17 (4.02)		
Placebo	PRI-S	Start	23.78 (10.27)	3.95 (4.28)	<0.001
		End	19.84 (8.47)		
	PRI-A	Start	1.59 (1.24)	0.27 (0.65)	0.796
		End	1.57 (1.19)		
	PRI-V	Start	1.54 (0.77)	0.081(0.64)	0.439
		End	1.46 (0.65)		
	PRI-TOTAL	Start	26.92 (11.12)	4.05 (4.50)	<0.001
		End	22.86 (9.33)		
	PPI	Start	1.7 (0.91)	0.35 (1.03)	0.056
		End	1.35 (0.59)		
	WORDS	Start	19.65 (10.56)	3.81 (4.78)	<0.001
		End	15.84 (8.95)		

Table created by authors. Comparison within each group before and after intervention across groups for the effective dimension (PRI-A). Main difference represents change from baseline to post-intervention. Abbreviations: total pain rating index (PRI-TOTAL), sensory pain rating index (PRI-S), affective pain rating index (PRI-A), evaluative pain rating index (PRI-V), present pain intensity (PPI); standard deviation (SD).

**Table 4:** Between-group comparisons for PRI-A.

Comparison	Mean Difference	95% CI	Cohen’s d
Experimental vs. Control	0.40	0.13–0.67	0.68
Experimental vs. Placebo	0.70	0.47–0.93	1.17

Table created by authors. Comparisons are reported for this secondary analysis (PRI-A). Abbreviations: CI, confidence interval; PRI-A, affective pain rating index.

## Data Availability

The data presented in this study are openly available in Zenodo at https://doi.org/10.5281/zenodo.19707767. The dataset has been fully anonymized to ensure participant confidentiality.

## Supplementary Materials

The following supporting information can be downloaded at https://www.mdpi.com/article/10.3390/medicina62081543/s1: File S1: CONSORT 2025 checklist. Dataset available at https://doi.org/10.5281/zenodo.19707767.

## Abbreviations

The following abbreviations are used in this manuscript:

ECA	Randomized clinical trial
MPQ	McGill Pain Questionnaire
TENS	Transcutaneous electrical nerve stimulation
PRI-A	Affective dimension
PRI-S	Sensory dimension
PRI-V	Evaluative dimension
PRI-TOTAL	Total pain rating index
PPI	Present pain intensity
CI	Confidence interval
